# Effects of Initial Lactate Levels and 24-h Lactate Clearance on Mortality in Post-Cardiac Arrest Patients: Insights from the Multicenter TIMECARD Registry

**DOI:** 10.7150/ijms.129084

**Published:** 2026-04-08

**Authors:** Hsin-Hui Hsu, Min-Shan Tsai, Li-Kuo Kuo, Chen-Hsu Wang, Chau-Chyun Sheu, Chun-Yu Wu, Shih-Ni Wu, Lung Chan, An-Yi Wang, Chew-Teng Kor

**Affiliations:** 1 Department of Critical Care Medicine, Changhua Christian Hospital, Changhua, Taiwan.; 2 Department of Emergency Medicine, College of Medicine National Taiwan University, Taipei, Taiwan.; 3 Department of Emergency Medicine National Taiwan University Hospital, Taipei, Taiwan.; 4 Department of Critical Care Medicine, MacKay Memorial Hospital, Taipei branch, Taiwan.; 5 Medical Intensive Care Unit, Cathay General Hospital, Taipei City, Taiwan.; 6 Division of Pulmonary and Critical Care Medicine, Kaohsiung Medical University Hospital, Kaohsiung, Taiwan.; 7 Department of Internal Medicine, Kaohsiung Medical University Hospital, Kaohsiung, Taiwan.; 8 School of Medicine, Graduate Institute of Medicine, College of Medicine, Kaohsiung Medical University, Kaohsiung, Taiwan.; 9 Division of Cardiology, Department of Internal Medicine, Ditmanson Medical Foundation Chia-Yi Christian Hospital, Chia-Yi, Taiwan; 10 Department of Emergency Medicine National Taiwan University Hospital, Taipei, Taiwan.; 11 Department of Neurology, Shuang Ho Hospital, Taipei Medical University, New Taipei City, Taiwan.; 12 Department of Neurology, School of Medicine, College of Medicine, Taipei Medical University, Taipei, Taiwan.; 13 Stroke Centre, Shuang Ho Hospital, Taipei Medical University, New Taipei City, Taiwan.; 14 Department of Emergency and Critical Medicine, Wan Fang Hospital, Taipei Medical University, Taipei, Taiwan.; 15 Department of Emergency Medicine, School of Medicine, College of Medicine, Taipei Medical University, Taiwan.; 16 Big Data and Digital Promotion Center, Changhua Christian Hospital, Changhua, 500, Taiwan.; 17 Graduate Institute of Statistics and Information Science, National Changhua University of Education, Changhua, 500, Taiwan.; 18 Graduate Institute of Clinical Medicine, College of Medicine, National Chung Hsing University, Taichung, Taiwan.

**Keywords:** lactate levels, lactate clearance, cardiac arrest, return of spontaneous circulation, Taiwan TIMECARD registry, mortality

## Abstract

Background: Lactate concentration and clearance are critical indicators of systemic hypoperfusion and can predict post-cardiac arrest outcomes. However, their combined prognostic value and factors influencing clearance achievement remain unexplored.

Methods: This retrospective study analyzed 1,016 adults with cardiac arrest from the Taiwan TIMECARD registry (January 2017-July 2024) who achieved return of spontaneous circulation (ROSC). Patients were stratified into quartiles based on initial lactate levels and 24-hour lactate clearance, defined as the percentage reduction in serum lactate from ROSC to 24 hours. Cox proportional hazards models were used to assess associations with 3-day and 30-day all-cause mortality following ROSC.

Results: Higher initial lactate levels (≥12.3 mmol/L) were associated with increased risks of 3-day (hazard ratio [HR]: 3.42, 95% confidence interval [CI]: 1.88-6.22) and 30-day mortality (HR: 1.88, 95% CI: 1.44-2.46). Poor lactate clearance (<16%) was associated with increased 3-day (HR: 21.05, 95% CI: 9.19-48.22) and 30-day mortality (HR: 2.74, 95% CI: 2.13-3.53). Patients achieving rapid clearance had better mortality outcomes despite moderately elevated initial levels, whereas those with both high initial lactate and poor clearance had a 6.9-fold higher 30-day mortality risk. Factors associated with low clearance included age ≥65 years, INR ≥1.15, Glasgow Coma Scale score ≤8, systolic blood pressure ≤125 mmHg, and repeat cardiopulmonary resuscitation within 1 h; conversely, targeted temperature management was protective.

Conclusion: Routine measurement of lactate levels at ROSC, together with serial monitoring after cardiac arrest, provides clinically meaningful prognostic information. Although initial lactate levels reflect disease severity, their prognostic impact may be modifiable through adequate clearance, whereas poor clearance is associated with an increased risk of mortality. Early identification of patients with inadequate clearance may facilitate targeted interventions to improve survival.

## Introduction

Cardiac arrest (CA), defined as the abrupt cessation of cardiac mechanical activity, remains a leading cause of mortality and morbidity worldwide.^1^,^2^ Restoration of spontaneous circulation (ROSC) is a primary objective of cardiopulmonary resuscitation (CPR).^3^ However, the post-ROSC period is often characterized by complex and evolving systemic disturbances, including cardiovascular instability, tissue hypoperfusion, and metabolic acidosis. Despite advancements in emergency response systems and resuscitation protocols, overall outcomes remain poor.^4^

The Pan-Asian Resuscitation Outcomes Study clinical research network reported substantial regional differences in out-of-hospital cardiac arrest (OHCA) epidemiology across Asia, with annual emergency medical services-treated OHCA incidence per 100,000 persons of 87.1 in Japan and 47.4 in Singapore. Notably, Seoul demonstrated the highest survival to hospital discharge (9.9%) and favorable neurologic recovery (3.7%).^5^ In Taiwan, in-hospital cardiac arrest (IHCA) incidence has declined by 70% from 2003 to 2020.^6^ Conversely, the incidence of OHCA is approximately 51.1 per 100,000 persons, with mortality rates varying over time and worse outcomes observed among older adults, women, and those with more comorbidities.^7^ In rural regions, ROSC rates are 19.7% and survival to emergency department discharge only 16.4%.^8^ These findings underscore the critical need for timely and high-quality post-cardiac arrest care.

Serum lactate concentration is a widely used marker of systemic hypoperfusion and cellular oxygen debt.^9^,^10^ During cardiac arrest, inadequate oxygen delivery coupled with anaerobic metabolism leads to systemic lactate accumulation. Lactate levels are widely used to assess the severity of circulatory failure^11^,^12^ predict post-cardiac arrest clinical outcomes.^13^-^16^ However, single-timepoint lactate measurements provide only an initial snapshot and may not fully reflect the evolving physiological status during the early critical hours following cardiac arrest. Lactate kinetics offer additional insights into circulatory recovery and organ perfusion following cardiac arrest. Higher rates of lactate clearance have been associated with improved survival outcomes.^17^,^18^ Han et al. identified that lactate clearance exceeding 34% within 6-h serves as a prognostic indicator of favorable outcomes during early post-cardiac arrest management.^19^ However, most existing studies have evaluated either lactate concentration or lactate clearance, but not both, in relation to post-cardiac arrest prognosis. A physiological continuum-based approach that integrates both parameters may provide a more comprehensive assessment of the severity and trajectory of post-cardiac arrest metabolic derangement. Additionally, no studies have investigated factors influencing lactate clearance rates.

In this study, we investigated the prognostic value of lactate kinetics in patients with ROSC following cardiac arrest. Specifically, we evaluated (1) the associations between lactate-related variables and mortality outcomes; (2) the combined prognostic value of lactate concentration and lactate clearance in predicting mortality risk; and (3) the factors influencing lactate clearance. We hypothesized that the combined assessment of lactate concentration and lactate clearance can be used to enhance patient-specific risk stratification in post-resuscitation care, thereby guiding clinicians in tailoring interventions to improve tissue perfusion and optimize patient outcomes.

## Materials and Methods

This retrospective cohort study utilized data from the Taiwan Management for Cardiac Arrest (TIMECARD) multicenter registry database, established by the Taiwan Society of Resuscitation and Critical Care (TSORCC) and described in detail previously.^20^-^22^ Briefly, 10 tertiary medical centers participated in the TSORCC collaborative network. The dataset included patients' baseline demographics, pre-existing comorbidities, location of cardiac arrest, resuscitation characteristics and medications, targeted temperature management (TTM) settings, extracorporeal membrane oxygenation (ECMO), documented DNR status, initial arrest rhythm, witnessed arrest, cause of arrest, laboratory data, vital signs, and Glasgow Coma Scale (GCS) scores. Survival status and date of death were also recorded.

We included 1,016 patients who experienced cardiac arrest with successful resuscitation and had at least two lactate measurements between January 2017 and July 2024. This study was conducted in accordance with the Declaration of Helsinki and was approved by the Institutional Review Board of Changhua Christian Hospital (No. 251202). Because the analyzed data were deidentified and encrypted, the requirement for informed consent was waived.

### Lactate-related variables

**Figure 1:**
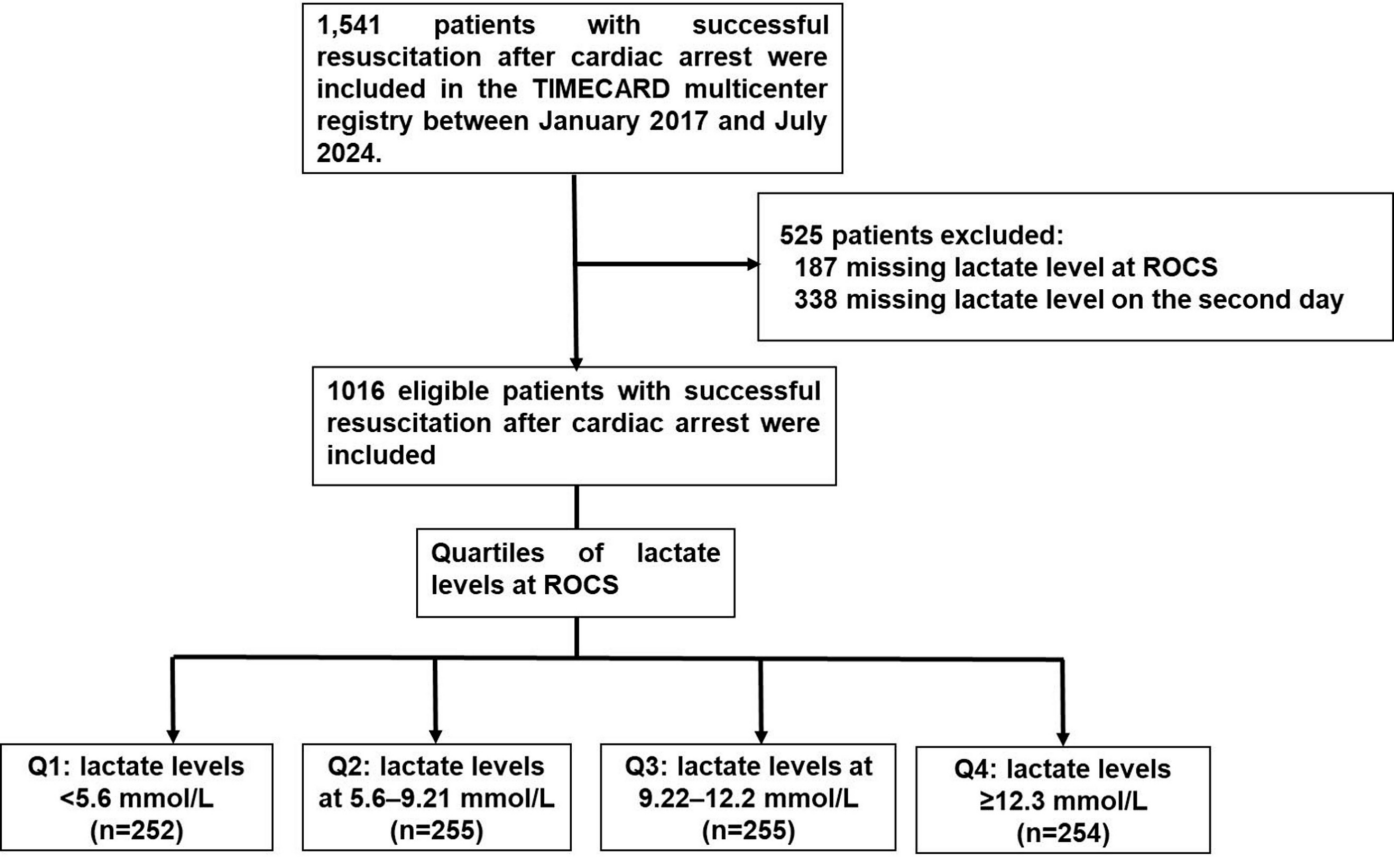
Study flow-chart.

The exposures of interest were serum lactate levels at ROSC and 24-hour lactate clearance. Initial lactate was defined as the first serum lactate measurement obtained at ROSC. Patients were stratified into quartiles based on their lactate levels at ROSC: Q1(< 5.6 mmol/L), Q2 (5.6-9.21 mmol/L), Q3 (9.22-12.2 mmol/L) and Q4 (≥ 12.3 mmol/L) (Figure 1).

Follow-up lactate measurement was defined as the lactate value recorded on post-ROSC day 1, which represents the closest clinically available measurement approximately 24 hours after ROSC. The 24-hours lactate clearance was calculated as the percentage change from lactate at ROSC to lactate at 24 hours using the following formula:

[(Lactate level at ROSC - Lactate level on post-ROSC day 1)/Lactate level at ROSC] × 100%

Patients were stratified into quartiles of 24-hour lactate clearance, with Q1 representing the highest clearance (≥ 72.8%), indicative of rapid and favorable lactate clearance, followed by Q2 (50%-72.7%), Q3 (16.0%-49.9%), and Q4 (< 16.0%), reflecting progressively poorer lactate clearance.

A risk matrix combining lactate levels at ROSC and 24-hour lactate clearance was constructed to evaluate 30-day mortality risk.

### Endpoints

The primary endpoints were 3-day and 30-day all-cause mortality. Patient death records were reviewed to ascertain mortality outcomes. Mortality follow-up was initiated at the time of return of spontaneous circulation (ROSC). Deaths following withdrawal of life-sustaining therapy were included as events. Patients were followed until death or the completion of the respective follow-up period (3-day or 30-day). Patients who were alive at the end of follow-up were censored at that time point.

### Statistical analysis

Categorical variables are presented as counts and percentages and were compared using the chi-square test. Continuous variables are expressed as mean ± standard deviation and were compared using one-way analysis of variance. Three lactate-related variables were examined: lactate level at ROSC, second-day lactate level, and 24-hour lactate clearance. Each variable was analyzed as both a continuous measure and as a categorical variable based on quartiles.

Associations between lactate-related variables and mortality outcomes were evaluated using Cox proportional hazards regression models. Crude hazard ratios (cHR) with 95% confidence intervals (95% CI) were obtained from unadjusted models, followed by adjusted hazard ratios (aHR) from models controlling for potential confounders. Subgroup analyses stratified by OHCA and IHCA were performed to assess cardiac arrest location effect modification. Kaplan-Meier survival curves were generated to visualize survival probabilities across lactate categories, and differences were evaluated using the log-rank test.

Logistic regression analysis was performed to identify factors associated with inadequate lactate clearance rates. Restricted cubic spline (RCS) functions were used to explore the relationships between continuous lactate-related variables and 30-day mortality and to identify cutoff values associated with achievement of target lactate clearance rates. Three-dimensional histograms and heatmaps were constructed to illustrate the combined effects of lactate level at ROSC and 24-hour lactate clearance on 30-day mortality.

All statistical analyses were performed using SAS software (version 9.4), and the visualization plot was performed using the “ggplot” package for R software in version 4.5.1. A two-sided p < 0.05 was considered statistically significant.

## Results

The mean age of the 1,016 included patients was 66.6±15.3 years, and their body mass index (BMI) was 24.4±5.4. Moreover, 352 (34.6%) and 664 (65.4%) experienced IHCA and OHCA, respectively. Overall, 158 (15.6%) patients died within 3 days and 530 (52.2%) died within 30 days (Table 1).

Table 1 lists the baseline characteristics of the patients with ROSC stratified by quartiles of lactate levels at ROSC: < 5.6 mmol/L (n = 252), 5.6-9.21 mmol/L (n = 255), 9.22-12.2 mmol/L (n = 255), and ≥ 12.3 mmol/L (n = 254) (Figure 1). Significant between-group differences were observed in baseline demographics and comorbidities. Compared with the other lactate quartiles, patients in the highest lactate quartile (≥ 12.3 mmol/L) were younger, had lower BMI, and had a lower prevalence of diabetes mellitus, hypertension, coronary artery disease, heart failure and end-stage renal disease on dialysis but had a higher prevalence of liver cirrhosis (all p < 0.05). IHCA predominated in the lowest lactate quartile (Q1: 52%), whereas OHCA was more frequent in highest quartile (Q4: 74.4%, p < 0.001). Overall, a cardiac etiology was identified in 51.97% of cardiac arrest cases, and a shockable rhythm was present in 41.93% of patients; however, neither differed significantly across lactate quartiles (p = 0.604 and 0.723, respectively). Ventricular tachycardia or ventricular fibrillation was observed in 30.51% of patients. Witnessed arrests were more common in Q1, accounting for 73.43% of cases.

Regarding resuscitation medications, the use of epinephrine and bicarbonate increased progressively with higher lactate levels (both p < 0.001). Lidocaine use was more common in Q3 (p = 0.038). CPR duration increased stepwise from 13.5 ± 12.2 min in the lowest lactate quartile to 27.7 ± 20.9 min in the highest (p < 0.001). Targeted temperature management (TTM) was most frequently applied in Q3 (55.3%), whereas extracorporeal membrane oxygenation (ECMO) was most commonly used in Q4 (25.2%).

Patients in higher lactate quartiles at ROSC exhibited lower systolic and diastolic blood pressures, body temperature, pH, and bicarbonate (HCO₃) as well as higher partial pressure of carbon dioxide (PCO₂), serum sodium, and serum potassium (all p < 0.01); these changes are consistent with more severe metabolic acidosis and hemodynamic instability.

Mortality rates rose progressively with lactate level: 3-day mortality increased from 6.7% in Q1 to 27.2% in Q4, and 30-day mortality increased from 37.7% to 67.3%, respectively (both p < 0.001).

### Association of lactate-related variables with mortality

**Figure 2:**
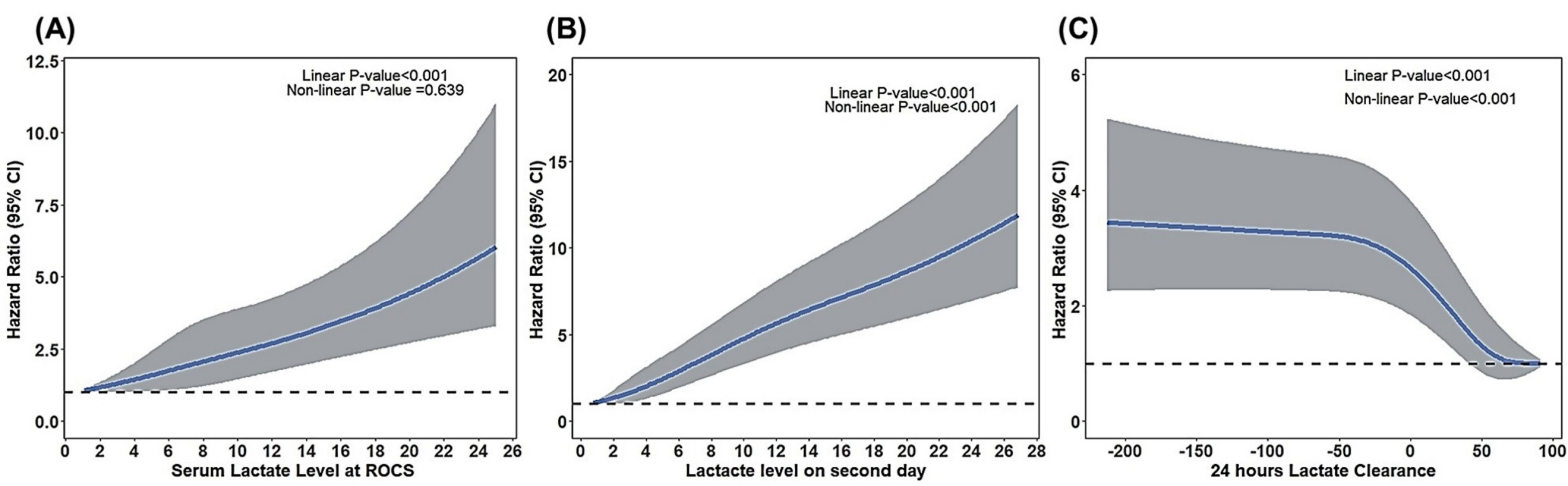
Restricted cubic spline showing the effects of lactate-related value on 30-day mortality.

In Figure 2, the RCS curve exhibits a continuous positive correlation between lactate-related variables and 30-day mortality. Higher lactate levels and lower lactate clearance were associated with an increased mortality risk. Notably, the 24-hour lactate clearance (Figure 2c) exhibited a nonlinear relationship: less or negative lactate clearance—indicating slower clearance or rising lactate levels—was linked to substantially higher mortality.

**Figure 3:**
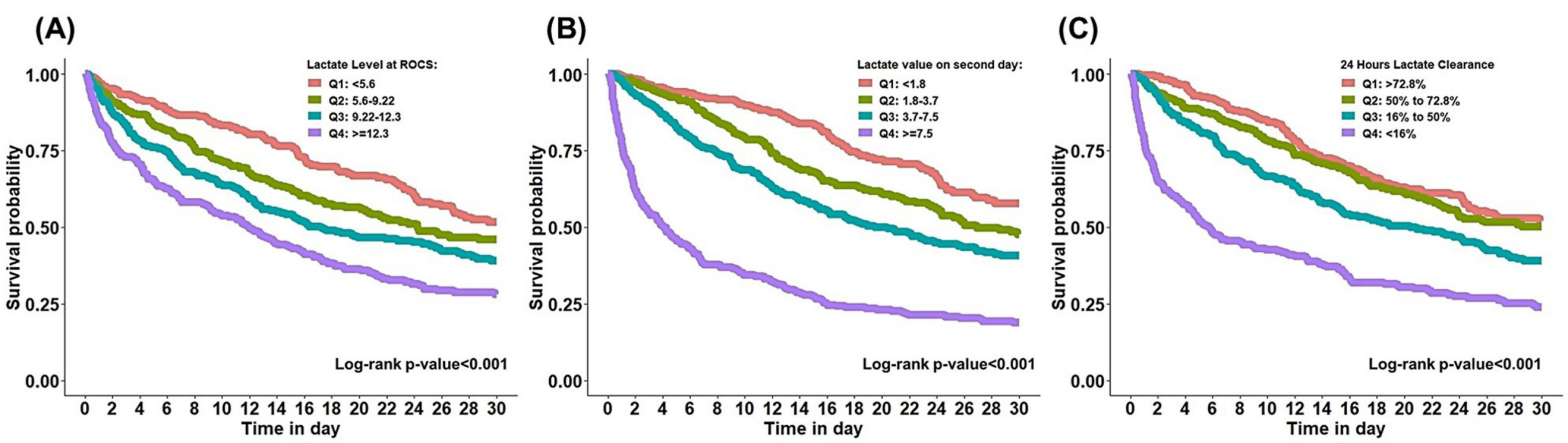
Kaplan-Meier curve for 30-day mortality based on quartiles of lactate-related categorical variables.

In Figure 3, Kaplan-Meier survival analysis revealed a clear separation of survival curves across lactate quartiles, demonstrating a significant association between higher lactate levels and reduced 30-day survival following cardiac arrest. Patients with the highest lactate at ROSC exhibited substantially lower survival throughout the 30-day follow-up period than the other groups. Similar patterns were observed for second-day lactate levels. Lactate clearance demonstrated a significant inverse association with survival; patients with inadequate lactate clearance (<50%) had markedly poorer survival outcomes than those achieving rapid clearance. The log-rank test confirmed significant differences in survival across lactate categories for all comparisons (p < 0.001).

Table 2 and Table 3 summarizes the results of multivariable Cox regression analysis of categorical lactate-related variables: lactate at ROSC, second-day lactate levels, and lactate clearance were independently associated with both 3-day and 30-day mortality (all p < 0.001).

Table 2 presents aHRs with 95% CI for 3-day mortality. Patients with lactate at ROSC ≥ 12.3 mmol/L had an approximately 3.4-fold higher adjusted hazard of death compared with the lowest category (< 5.6 mmol/L) (aHR: 3.42, 95% CI: 1.88-6.22, p < 0.001). Intermediate categories also displayed an elevated risk, with aHR of 1.65 (95% CI: 0.89-3.04) for 5.6-9.1 mmol/L and 1.98 (95% CI: 1.09-3.57) for 9.22-12.2 mmol/L. Second-day lactate ≥ 7.5 mmol/L was associated with markedly higher mortality risk (aHR: 15.76, 95% CI: 7.87-31.55, p < 0.001), whereas intermediate second-day lactate levels (3.7-7.4 mmol/L) also conferred increased risk (aHR: 2.97, 95% CI: 1.37-6.43, p = 0.006). Lactate clearance < 16% was associated with substantially elevated short-term mortality risk (aHR: 21.05, 95% CI: 9.19-48.22, p < 0.001). Intermediate clearance categories also exhibited an elevated risk, with aHRs of 5.65 (95% CI: 2.35-13.55) and 3.44 (95% CI: 1.38-8.57) for clearance of 16.0%-49.9% and 50%-72.7%, respectively, compared with optimal clearance rate at ≥ 72.8%.

For 30-day mortality in Table 3, lactate at ROSC ≥ 12.3 mmol/L was associated with an aHR of 1.88 (95% CI: 1.44-2.46, p < 0.001). Second-day lactate ≥ 7.5 mmol/L conferred an aHR of 3.83 (95% CI: 2.88-5.07, p < 0.001). Lactate clearance < 16% remained associated with increased mortality risk (aHR: 2.74, 95% CI: 2.13-3.53, p < 0.001).

Across both outcomes, the results supported the prognostic importance of high initial lactate, elevated second-day lactate, and unfavorable lactate kinetics. Greater lactate clearance was linked to improved survival. No significant interactions were observed between OHCA and IHCA subgroups, except that patient with OHCA with second-day lactate ≥ 7.5 mmol/L demonstrated greater 30-day mortality risk than patients with IHCA.

### Combined effects of lactate-related variables and 30-day mortality

Mortality risk varied across different combinations of initial lactate levels at ROSC and 24-h lactate clearance, with a significant interaction between the two variables (P for interaction < 0.001). Using the lowest quartile (Q1) of both lactate at ROSC and lactate clearance as the reference category, distinct patterns emerged based on clearance rates.

**Figure 4:**
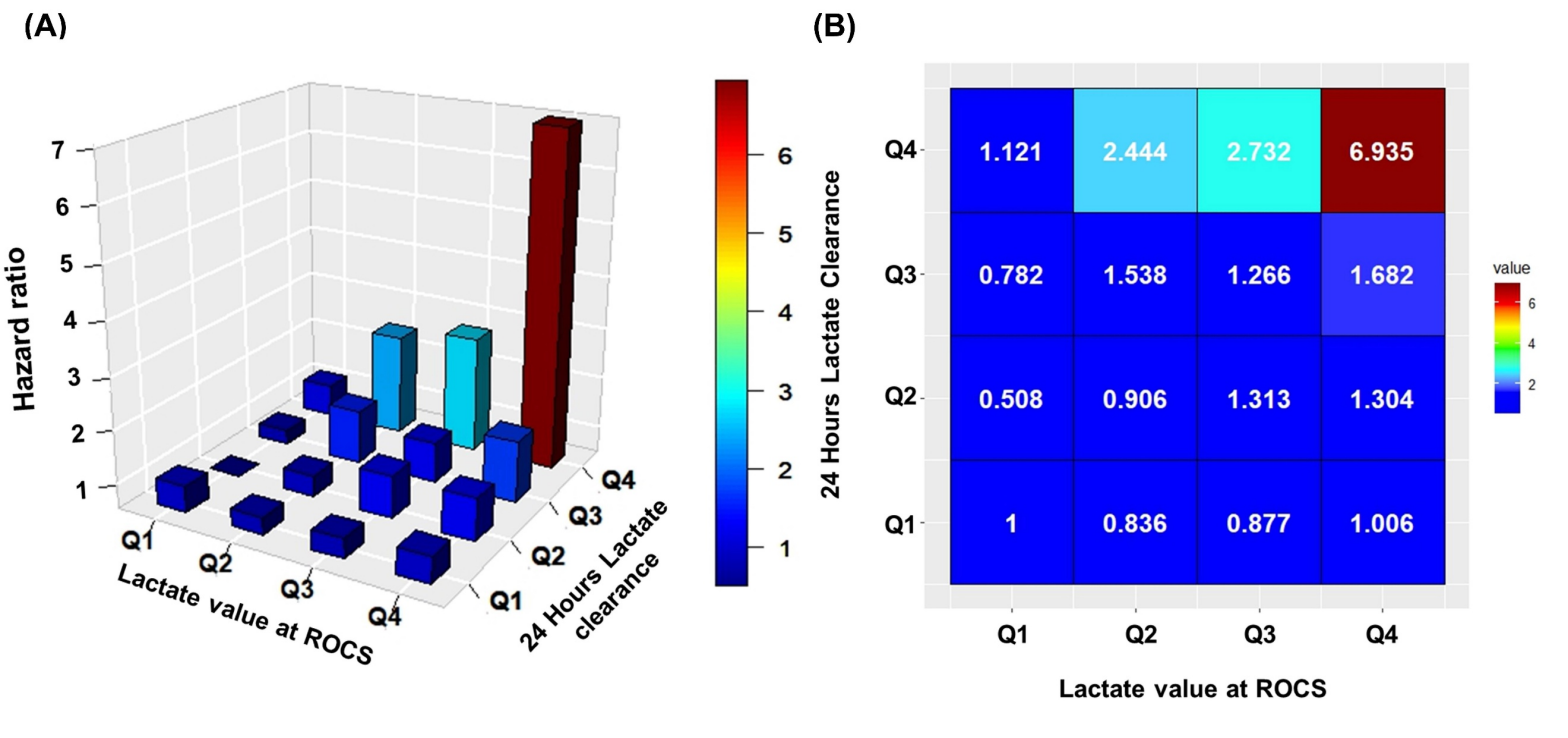
Three-dimensional histogram and heatmap showing the hazard ratio of lactate value at ROCS and 24-h lactate clearance on 30-day mortality.

In Figure 4, patients with lactate clearance > 72.8% demonstrated favorable mortality outcomes across all baseline lactate levels at ROSC (HR: 0.836-1.006), indicating that robust early lactate clearance is protective regardless of initial lactate burden.

For patients with suboptimal clearance (50%-72.7%), favorable outcomes were limited to those with low baseline lactate levels, as evidenced by an HR of 0.508 for Q1 (< 5.6 mmol/L) and 0.906 for Q2 (5.6-9.21 mmol/L). Conversely, patients with higher baseline lactate levels (Q3 and Q4) experienced substantially increased mortality risk (HR: 1.313-1.304).

At moderate clearance rate (Q3: 16.0%-49.9%), mortality outcomes again depended on baseline lactate levels, as evidenced by a low HR of 0.782 for Q1. However, those with higher baseline lactate (Q2-Q4) experienced progressively increased mortality hazard (HR: 1.226-1.682). These results indicated that moderate clearance is inadequate for managing elevated initial lactate.

Poor or negative lactate clearance combined with high baseline lactate was associated with the greatest mortality risk. Patients with high lactate at ROSC (≥ 12 mmol/L) and poor clearance or ongoing lactate accumulation demonstrated a 6.9-fold increased hazard of 30-day mortality (HR: 6.938).

These findings indicate that patients with higher lactate levels at ROSC who achieved substantial clearance (> 73%) within 24 hours demonstrated lower mortality risk, whereas those with low lactate levels at ROSC showed favorable outcomes even with lower clearance rates. Inadequate clearance relative to initial lactate burden was associated with progressively increased 30-day mortality risk.

### Factors contributing to inadequate and poor lactate clearance

**Figure 5:**
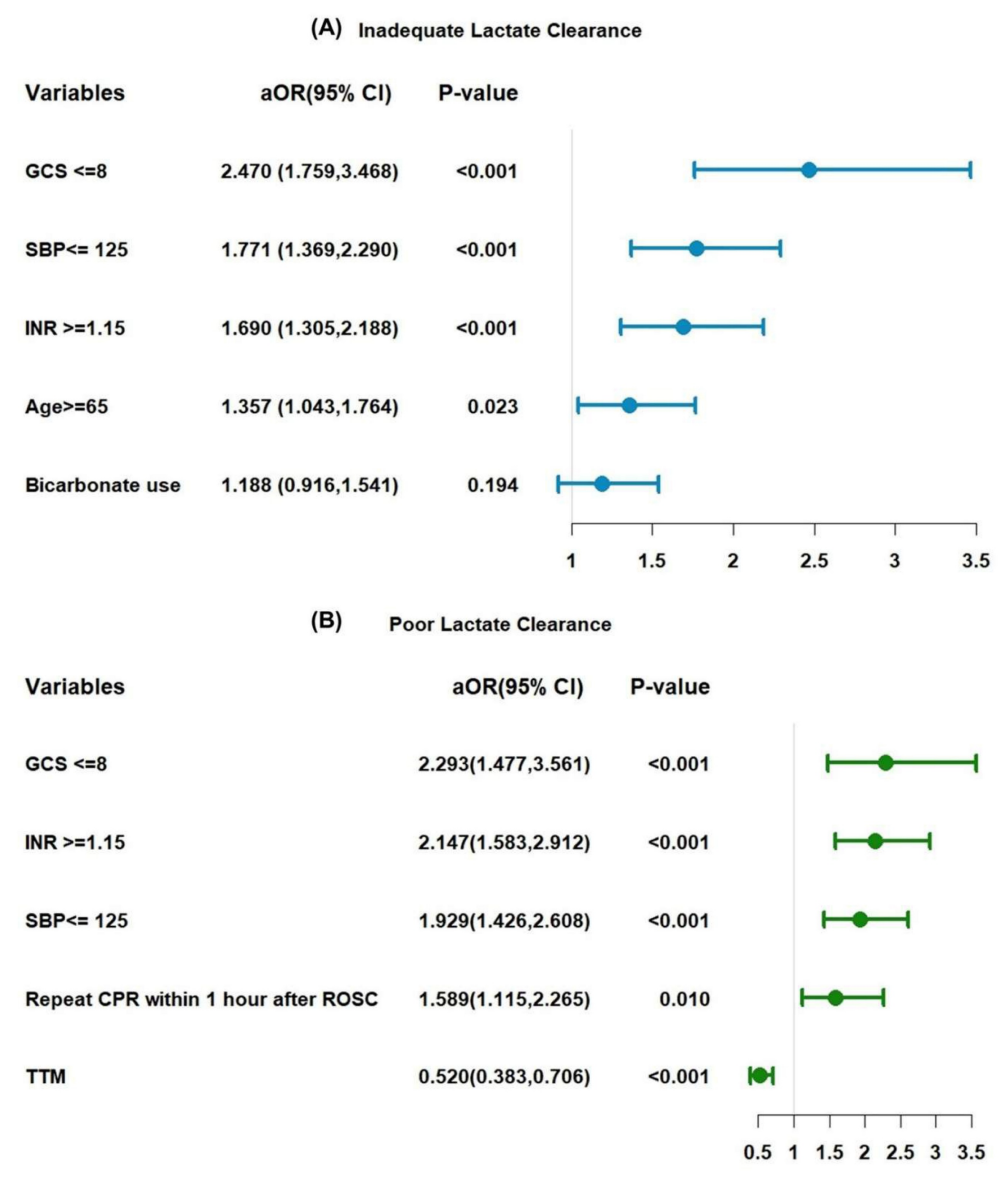
Risk factor of inadequate clearance and poorly lactate clearance.

Multivariate logistic regression analysis revealed four variables significantly associated with inadequate lactate clearance (< 50%): age ≥ 65 years (adjusted odds ratio [aOR]: 1.357; 95% CI: 1.043-1.764; p = 0.023), INR ≥ 1.15 (aOR: 1.690; 95% CI: 1.305-2.188; p < 0.001), GCS score ≤ 8 (aOR: 2.470; 95% CI: 1.759-3.468; p < 0.001), and systolic blood pressure (SBP) ≤ 125 mmHg (aOR: 1.771; 95% CI: 1.369-2.290; p < 0.001). Bicarbonate use was not statistically significant (aOR: 1.188; 95% CI: 0.916-1.541; p = 0.194) (Figure 5A).

Figure 5B presents the results for poor lactate clearance, defined as a clearance rate of < 16%. Significant predictors included GCS score ≤ 8 (aOR: 2.293; 95% CI: 1.477-3.561; p < 0.001), INR ≥ 1.15 (aOR: 2.147; 95% CI: 1.583-2.912; p < 0.001), and SBP ≤ 125 mmHg (aOR: 1.929; 95% CI: 1.426-2.608; p < 0.001). Additionally, repeat CPR within 1-h after ROSC increased the likelihood of poor lactate clearance (aOR: 1.589; 95% CI: 1.115-2.265; p = 0.010), whereas TTM was a protective factor (aOR: 0.520; 95% CI: 0.383-0.706; p < 0.001). The cutoff points for each factor were determined using RCS analysis (Figure S1).

## Discussion

Our analysis of a large, multicenter registry of patients after cardiac arrest revealed that absolute lactate levels at ROSC, second-day lactate levels, and 24-h lactate clearance were independently associated with both 3-day and 30-day mortality. The highest mortality rates were observed in patients with lactate at ROSC ≥ 12.3 mmol/L or second-day lactate ≥ 7.5 mmol/L. Furthermore, unfavorable lactate kinetics—reflected by inadequate or absent lactate decline within 24 h—substantially amplified mortality risk. Combined analysis revealed that patients with higher initial lactate levels who achieved outcomes comparable to those with lower initial lactate demonstrated progressively greater clearance rates. Among clinicodemographic variables, older age, higher INR, lower GCS score, lower SBP, repeated CPR within 1 h after ROSC, and bicarbonate use were significantly associated with poor lactate clearance, whereas TTM was associated with improved lactate clearance.

The strong association between elevated lactate levels and mortality likely reflects the combined effects of systemic hypoperfusion, impaired oxygen utilization, and metabolic derangements during and after resuscitation^23^-^25^. High lactate at ROSC indicates prolonged low-flow states, delayed clearance, or ongoing tissue hypoxia, all of which are markers of severe post-cardiac arrest syndrome^26^-^28^. Serum lactate > 4 mmol/L is generally considered a threshold for increased mortality^26^,^29^-^32^. However, no consensus exists regarding the optimal lactate range for patients after cardiac arrest, as it varies by the timing of measurement and patient population. In IHCA, mortality risk increases with lactate levels: 44% for < 5 mmol/L, 58% for 5-10 mmol/L, and 73% for > 10 mmol/L^33^. In nontraumatic OHCA, lactate cutoffs of 9.1 and 9.4 mmol/L predict 24-h and 48-h mortality, respectively^34^. In another multicenter OHCA cohort, survival dropped sharply when lactate exceeded 10.6 mmol/L^35^. Consistently, our findings demonstrate a linear positive relationship between lactate at ROSC and 30-day mortality, with initial lactate of < 5.6 mmol/L and second-day lactate of < 3.7 mmol/L associated with better survival.

Rapid lactate clearance reflects effective restoration of tissue oxygenation and resolution of anaerobic metabolism, whereas persistently high or increasing lactate levels suggest ongoing hypoperfusion, mitochondrial dysfunction, or continued metabolic stress. Inadequate clearance strongly predicts poor outcomes^18^,^28^,^36^. Specifically, failure to achieve significant reduction within 24-hour, such as not reaching thresholds of 1.55-3.1 mmol/L, and 34% or <65% clearance at 6 h has been linked to poor survival^18^,^36^-^38^. Our results are consistent, showing that clearance greater than 72.8% within 24-hour is associated with improved survival. While early lactate clearance at 6 hours primarily reflects acute resuscitation adequacy and guides immediate interventions, 24-hour clearance may better capture sustained metabolic recovery and identify patients at risk for delayed deterioration despite initial hemodynamic stabilization.

Our findings demonstrate that baseline lactate and clearance are not independent prognostic markers but a significant interplay component of a physiologic continuum, thus providing a more comprehensive risk assessment than isolated measurements. The association between lactate clearance and mortality was modified by initial lactate levels. Specifically, patients with low baseline lactate (< 5.6 mmol/L) showed favorable outcomes across a range of clearance rates, whereas those with high baseline lactate (≥ 12.3 mmol/L) with clearance > 72.8% within 24-hour associated with mortality rates comparable to lower-risk patients. These observations may inform risk stratification and clinical decision-making. Patients with high initial lactate and inadequate clearance represent a high-risk subset who may warrant closer monitoring and thorough assessment of resuscitation adequacy. However, given the observational nature of this study, we cannot establish causality between lactate clearance and improved survival. We do not claim that interventions specifically targeting a clearance threshold of ≥ 72.8% will directly improve clinical outcomes. Instead, lactate clearance likely acts as a surrogate marker of global physiological recovery in response to appropriate resuscitation, rather than serving as an independent therapeutic target. Future prospective trials are needed to evaluate whether lactate clearance-guided protocols improve clinical outcomes compared to standard care. The limited effect of bicarbonate administration implies that buffering alone is insufficient^39^; instead, treatment should focus on underlying causes of hyperlactatemia, optimizing cardiac output, improving perfusion, enhancing oxygen delivery, and correcting metabolic derangements.

We identified several factors associated with low lactate clearance (age ≥ 65 years, INR ≥ 1.15, GCS score ≤ 8, SBP ≤ 125 mmHg, and repeat CPR within 1 hour after ROSC), which may help clinicians recognize patients at risk of impaired metabolic recovery and guide targeted interventions. Gissel et al. demonstrated that coagulation dynamics are significantly impaired when pH falls to levels typical of metabolic acidosis in severe trauma. Inadequate tissue perfusion leads to hypoxia, anaerobic metabolism, and lactic acid accumulation, and the resulting acidosis inhibits key coagulation enzymes, exacerbating coagulopathy^40^. In our study, the mean pH was 7.1 ± 0.2, indicating clinically significant acidosis, which likely contributed to both impaired coagulation and reduced lactate metabolism. Impaired perfusion not only promotes lactate buildup but also hinders its clearance, as demonstrated by He et al. in patients with sepsis^41^. In our cohort, elevated INR, which is a marker of coagulopathy, may similarly reflect impaired tissue perfusion and oxygen delivery, linking coagulation dysfunction to reduced lactate clearance. High INR may therefore serve as a biomarker for identifying patients at risk of poor lactate clearance; further research is warranted to comprehensively investigate this relationship.

Although TTM does not directly reduce lactate levels, it stabilizes metabolic processes disrupted by ischemia, improves glucose metabolism, and reduces lactate production. TTM may also enhance organ perfusion by stabilizing the blood-brain barrier and mitigating endothelial dysfunction^42^, thereby creating favorable conditions for recovery and promoting lactate clearance. In our cohort, TTM was significantly associated with improvement in mild lactate clearance (< 16%) but had no effect in cases with moderate reductions (< 50%), suggesting that TTM should be considered a supportive strategy rather than a primary intervention for lactate elimination.

The strengths of this study include the use of a large, multicenter registry database; integrated assessment of multiple lactate-related variables; adjustment for key confounders; and identification of factors associated with poor clearance. The study also had some limitations. First, this was a retrospective observational study, which precludes the establishment of causal relationships. Second, the TIMECARD registry does not capture data on arrest downtime, CPR quality, or the timing of DNR documentation relative to cardiac arrest, all of which may reflect underlying illness severity and influence goals of care decisions. Third, unmeasured post-ROSC interventions—including vasopressor strategies, fluid management, hemodynamic optimization protocols, and institutional practice variations—represent potential sources of residual confounding that may influence the observed associations. Finally, the identified lactate thresholds should be regarded as hypothesis-generating, intended to inform risk stratification rather than define fixed treatment targets. Future studies should prospectively validate both the conceptual framework and specific numerical thresholds across diverse healthcare settings with varying prehospital response capabilities, post-cardiac arrest care protocols, and TTM practices before these values are adopted into clinical practice.

## Conclusion

Our results support the use of serial lactate measurement from ROSC through the early post-resuscitation period to provide meaningful prognostic information after cardiac arrest. Importantly, the prognostic impact of 24-hour lactate clearance dependent on the initial lactate level at ROSC. Patients with moderately to highly elevated lactate levels at ROSC demonstrated a strong association between higher 24-hour lactate clearance and more favorable outcomes, whereas poor clearance was associated with markedly increased risk of mortality. These findings establish lactate clearance as a baseline-adjusted prognostic biomarker that provides dynamic information regarding physiological recovery during post-cardiac arrest care, particularly in patients with marked metabolic derangement. Lactate clearance reflects the adequacy of resuscitative efforts aimed at restoring tissue perfusion and cellular oxygen delivery, rather than serving as a direct therapeutic target. Early identification of patients with high lactate levels and insufficient clearance may assist in risk stratification, inform decisions regarding the intensity of ongoing supportive care, and help assess whether resuscitative interventions are achieving their intended physiological effect during the post-cardiac arrest care continuum.

## Supplementary Material

Supplementary figure.

## Figures and Tables

**Table 1:** Patients demographic and clinical variables

	Lactate value at the time of ROSC				Total	P-value
	Q1: < 5.6	Q2:5.6- < 9.22	Q3:9.22- < 12.3	Q4: ≥ 12.3		
Sample size	252	255	255	254	1016	
Age	68.1±15.2	66.7±14.9	67.2±15	64.5±15.8	66.6±15.3	0.047
Male	171 (67.9%)	185 (72.5%)	152 (59.6%)	173(68.1%)	681 (67%)	0.018
BMI	23.9±5.2	25.2±5.3	24.7±5.3	24.1±5.6	24.4±5.4	0.028
Comorbidity disease						
Diabetes mellitus	117 (46.4%)	109 (42.7%)	111 (43.5%)	85 (33.5%)	422 (41.5%)	0.020
Hypertension	170 (67.5%)	158 (62%)	166 (65.1%)	134 (52.8%)	628 (61.8%)	0.004
Coronary artery disease	80 (31.7%)	67 (26.3%)	79 (31%)	39 (15.4%)	265 (26.1%)	< 0.001
Heart failure	49 (19.4%)	41 (16.1%)	34 (13.3%)	26 (10.2%)	150 (14.8%)	0.026
Valvular heart disease	11 (4.4%)	9 (3.5%)	13 (5.1%)	8 (3.1%)	41 (4%)	0.683
Arrhythmia	47 (18.7%)	36 (14.1%)	31 (12.2%)	33 (13%)	147 (14.5%)	0.162
COPD	27 (10.7%)	21 (8.2%)	23 (9%)	21 (8.3%)	92 (9.1%)	0.743
Chronic kidney disease	45 (17.9%)	41 (16.1%)	43 (16.9%)	26 (10.2%)	155 (15.3%)	0.075
ESRD	41 (16.3%)	34 (13.3%)	36 (14.1%)	17 (6.7%)	128 (12.6%)	0.008
Liver cirrhosis	11 (4.4%)	6 (2.4%)	4 (1.6%)	17 (6.7%)	38 (3.7%)	0.011
Cerebral vascular accident	35 (13.9%)	40 (15.7%)	32 (12.5%)	38 (15%)	145 (14.3%)	0.762
Dementia	25 (9.9%)	10 (3.9%)	19 (7.5%)	17 (6.7%)	71 (7%)	0.068
Bedridden	27 (10.7%)	18 (7.1%)	17 (6.7%)	21 (8.3%)	83 (8.2%)	0.339
Post tracheostomy	7 (2.8%)	3 (1.2%)	2 (0.8%)	2 (0.8%)	14 (1.4%)	0.170
Hyperlipidemia	43 (17.1%)	48 (18.8%)	30 (11.8%)	44 (17.3%)	165 (16.2%)	0.148
Malignancy	42 (16.7%)	45 (17.6%)	45 (17.6%)	51 (20.1%)	183 (18%)	0.780
CA location						
INCA	131 (52%)	82 (32.2%)	74 (29%)	65 (25.6%)	352 (34.6%)	< 0.001
OHCA	121 (48%)	173 (67.8%)	181 (71%)	189 (74.4%)	664 (65.4%)	
CPR medication						
Epinephrine	204 (81%)	210 (82.4%)	228 (89.4%)	237 (93.3%)	879 (86.5%)	< 0.001
Amiodarone	45 (17.9%)	55 (21.6%)	57 (22.4%)	52 (20.5%)	209 (20.6%)	0.617
Lidocaine	16 (6.3%)	19 (7.5%)	32 (12.5%)	17 (6.7%)	84 (8.3%)	0.038
Calcium gluconate	26 (10.3%)	18 (7.1%)	34 (13.3%)	33 (13%)	111 (10.9%)	0.084
Calcium chloride	16 (6.3%)	34 (13.3%)	31 (12.2%)	28 (11%)	109 (10.7%)	0.060
Bicarbonate	83(32.9%)	98 (38.4%)	107 (42%)	149 (58.7%)	437 (43%)	< 0.001
Initial electrocardiogram rhythm						
Asystole	37 (14.68%)	52 (20.39%)	69 (27.06%)	70 (27.56%)	228 (22.44%)	0.011
Pulseless Electrical Activity	108 (42.86%)	85 (33.33%)	79 (30.98%)	90 (35.43%)	362 (35.63%)	
Ventricular Tachycardia or Fibrillation	80 (31.75%)	87 (34.12%)	76 (29.8%)	67 (26.38%)	310 (30.51%)	
Other	27 (10.71%)	31 (12.16%)	31 (12.16%)	27 (10.63%)	116 (11.42%)	
Shockable rhythm	99 (39.29%)	110 (43.14%)	112 (43.92%)	105 (41.34%)	426 (41.93%)	0.723
Cause of cardia arrest						
non-cardiac	119 (47.22%)	117 (45.88%)	121 (47.45%)	131 (51.57%)	488 (48.03%)	0.604
cardiac	133 (52.78%)	138 (54.12%)	134 (52.55%)	123 (48.43%)	528 (51.97%)	
Witnessed arrest	203 (80.56%)	184 (72.16%)	173 (67.84%)	186 (73.23%)	746 (73.43%)	0.013
TTM use	103 (40.9%)	139 (54.5%)	141 (55.3%)	112 (44.1%)	495 (48.7%)	0.001
ECMO	22 (8.73%)	42 (16.47%)	57 (22.35%)	64 (25.2%)	185 (18.21%)	< 0.001
DNR status	116 (46.03%)	130 (50.98%)	148 (58.04%)	144 (56.69%)	538 (52.95%)	0.025
CPR duration	13.5±12.2	21±17.8	22.9±17.7	27.7±20.9	21.3±18.1	< 0.001
Vital sign at ROSC						
Heart rate	103.1±31.6	104.4±31	103.6±29.8	108.2±29.3	104.8±30.5	0.218
SBP	135.5±40.2	132.6±42.3	127.7±44.6	119.4±40.5	128.8±42.3	< 0.001
DBP	77.1±24	78±27.1	73.9±26.7	70.3±27	74.8±26.4	0.004
Body temperature	36.2±1.3	36±1.3	35.8±1.2	35.5±1.4	35.9±1.3	< 0.001
Lab data at ROSC						
PCO_2_	57.5±29.4	59±26.6	68.7±30.9	75.7±35.8	65.2±31.7	< 0.001
PO_2_	112.9±122.2	107.4±117.2	103.5±114.5	99.6±109.7	105.9±115.9	0.610
HCO_3_	21.3±6.5	18.4±5.1	16.9±4.7	14.4±6	17.7±6.2	< 0.001
WBC count	12980±7229	14209±7165	13120±6074	14952±9089	13816±7500	0.008
Hemoglobin	11.7±3	11.9±3.1	11.9±3.1	11.7±3.5	11.8±3.2	0.772
Platelet count	226.2±107	1153±14727	210±102	210±104	451±7379	0.379
INR	1.2±0.3	1.2±0.6	1.3±0.5	1.5±1.4	1.3±0.8	< 0.001
Creatinine	2.7±2.8	2.4±2.3	2.5±2.6	2±1.5	2.4±2.4	0.002
Sodium	136.9±7.1	136±6.8	137±7	138.7±7.5	137.2±7.1	< 0.001
Potassium	4.4±1.3	4.4±1.3	4.5±1.3	4.9±1.4	4.5±1.3	< 0.001
pH	7.2±0.2	7.1±0.2	7±0.2	7±0.2	7.1±0.2	< 0.001
Sustained ROSC	211 (83.7%)	205(80.4%)	214 (83.9%)	211 (83.1%)	841 (82.8%)	0.699
GCS at ROSC	7.3±4	7.2±3.7	6.9±3.3	6.2±3.0	6.9±3.6	0.002
Lactate value at ROSC	3.6±1.3	7.4±1.0	10.6±0.8	16.1±3.7	9.5±5.0	< 0.001
Lactate value on day 1	3.1±3.3	4.0±3.2	6.2±4.8	9.9±8.3	5.8±5.9	< 0.001
24-h lactate clearance	25.2±47.9	48.2±37.1	41.8±44.2	41.2±43.1	39.3±43.9	< 0.001
Mortality outcome						
3-day mortality	17 (6.7%)	30 (11.8%)	42 (16.5%)	69 (27.2%)	158 (15.6%)	< 0.001
30-day mortality	95 (37.7%)	119 (46.7%)	145 (56.9%)	171 (67.3%)	530 (52.2%)	< 0.001

Abbreviations: COPD, chronic obstructive pulmonary disease; ESRD, end-stage renal disease; CA, cardiac arrest; IHCA, in-of-hospital cardiac arrest; OHCA, out-of-hospital cardiac arrest; CPR, cardiopulmonary resuscitation; ROSC, return of spontaneous circulation; TTM, targeted temperature management; ECMO, extracorporeal membrane oxygenation; DNR, do-not-resuscitate; SBP, systolic blood pressure; DBP, diastolic blood pressure; WBC, white blood cell; INR, International Normalized Ratio; GCS, Glasgow Coma Scale scores

**Table 2:** The hazard ratio of lactate-related variables as a categorical variable based on quartiles on 3-day mortality

Lactate-related variables	Cardio-arrest patients (n=1016)				OHCA	IHCA	P_interaction_
	cHR (95% CI)	P-value	aHR (95% CI)	P-value	aHR (95% CI)	aHR (95% CI)	
lactate value at the time of ROSC							
Q1:< 5.6	1		1		1	1	
Q2:5.6 - < 9.22	1.78 (0.98,3.22)	0.058	1.65(0.89,3.04)	0.1093	1.94 (0.81,4.62)	1.52 (0.65,3.56)	0.700
Q3:9.22 - < 12.3	2.55 (1.45,4.48)	0.001	1.98(1.09,3.57)	0.0241	1.73 (0.71,4.24)	2.37 (1.07,5.25)	0.604
Q4:≥ 12.3	4.59 (2.70,7.81)	< 0.001	3.42(1.88,6.22)	<. 0001	2.88 (1.23,6.77)	3.67 (1.69,8.01)	0.680
Lactate value on day 1							
Q1:< 1.8	1		1		1	1	
Q2: 1.8 - < 3.7	1.23 (0.51,2.97)	0.646	1.30(0.54,3.16)	0.560	3.20 (0.48,21.22)	0.93 (0.34,2.52)	0.257
Q3:3.7 - < 7.5	2.74 (1.27,5.90)	0.010	2.97(1.37,6.43)	0.006	4.56 (0.69,30.32)	2.26 (1.00,5.09)	0.503
Q4: ≥ 7.5	17.11 (8.67,33.74)	< 0.001	15.76(7.87,31.55)	< 0.001	42.98 (8.04,229.59)	9.36 (4.49,19.51)	0.101
Lactate clearance							
Q1≥ 72.8%	1		1		1	1	
Q2:50% - < 72.8%	3.43 (1.38,8.55)	0.008	3.44(1.38,8.57)	0.008	6.18 (0.30,128.5)	3.03 (1.22,7.54)	0.659
Q3:16.0% - < 50%	5.56 (2.33,13.3)	< 0.001	5.65(2.35,13.55)	< 0.001	14.88 (0.78,283.03)	4.24 (1.78,10.13)	0.424
Q4:< 16.0%	21.78 (9.55,49.65)	< 0.001	21.05(9.19,48.22)	< 0.001	78.00 (4.47,1360.75)	12.64 (5.58,28.64)	0.230

Abbreviations: IHCA, in-of-hospital cardiac arrest; OHCA, out-of-hospital cardiac arrest; cHR, crude hazard ratio; aHR, adjusted hazard ratio; CI, confidence interval; aHRs were estimated using multivariable Cox proportional hazards regression models with a backward elimination procedure. In step 1, the models were adjusted for potential confounders, including age, sex, BMI, comorbid conditions, OHCA/INCA, CPR medications, vital signs at ROSC, laboratory data at ROSC, resuscitation characteristics (sustained ROSC, CPR duration, cause of cardiac arrest, initial electrocardiogram rhythm, shockable rhythm, and witnessed arrest), GCS score, post-resuscitation interventions (TTM and ECMO), and do-not-resuscitate (DNR) status

**Table 3:** The hazard ratio of lactate-related variables as a categorical variable based on quartiles on 30-day mortality

Lactate-related variables	Cardio-arrest patients (n=1016)					OHCA	IHCA	P_interaction_
	cHR (95% CI)	P-value	aHR (95% CI)	P-value	aHR (95% CI)		aHR (95% CI)	
Lactate value at the time of ROSC								
Q1:< 5.6	1		1		1		1	
Q2:5.6 - < 9.22	1.36 (1.03,1.77)	0.027	1.41 (1.07,1.85)	0.015	1.72 (1.12,2.64)		1.18 (0.82,1.70)	0.191
Q3:9.22 - < 12.3	1.70 (1.32,2.21)	< 0.001	1.59 (1.22,2.07)	< 0.001	1.49 (0.97,2.28)		1.53 (1.08,2.17)	0.919
Q4:≥12.3	2.42 (1.88,3.11)	< 0.001	1.88 (1.44,2.46)	< 0.001	2.23 (1.45,3.42)		1.62 (1.14,2.3)	0.252
Lactate value on day 1								
Q1:< 1.8	1		1		1		1	
Q2: 1.8 - < 3.7	1.51 (1.13,2.03)	0.006	1.41 (1.04,1.89)	0.025	1.88 (1.14,3.12)		1.21 (0.84,1.76)	0.167
Q3:3.7 - < 7.5	2.02 (1.53,2.68)	< 0.001	1.66 (1.24,2.21)	< 0.001	1.85 (1.09,3.12)		1.54 (1.09,2.17)	0.572
Q4: ≥7.5	4.92 (3.77,6.42)	< 0.001	3.83 (2.88,5.07)	< 0.001	6.15 (3.76,10.08)		2.88 (2.06,4.02)	0.011
Lactate clearance								
Q1≥72.8%	1		1		1		1	
Q2:50% - < 72.8%	1.13 (0.86,1.49)	0.379	1.14 (0.86,1.51)	0.358	1.33 (0.79,2.26)		1.08 (0.77,1.50)	0.495
Q3:16.0% - < 50%	1.59 (1.22,2.06)	0.001	1.49 (1.15,1.93)	0.003	1.68 (1.00,2.82)		1.42 (1.05,1.92)	0.582
Q4:< 16.0%	3.05 (2.38,3.91)	< 0.001	2.74 (2.13,3.53)	< 0.001	3.15 (1.96,5.07)		2.66 (1.97,3.59)	0.548
								

Abbreviations: IHCA, in-of-hospital cardiac arrest; OHCA, out-of-hospital cardiac arrest; cHR, crude hazard ratio; aHR, adjusted hazard ratio; CI, confidence interval; aHRs were estimated using multivariable Cox proportional hazards regression models with a backward elimination procedure. In step 1, the models were adjusted for potential confounders, including age, sex, BMI, comorbid conditions, OHCA/INCA, CPR medications, vital signs at ROSC, laboratory data at ROSC, resuscitation characteristics (sustained ROSC, CPR duration, cause of cardiac arrest, initial electrocardiogram rhythm, shockable rhythm, and witnessed arrest), GCS score, post-resuscitation interventions (TTM and ECMO), and do-not-resuscitate (DNR) status.

## Data Availability

The data underlying this article are available from the TIMECARD database but cannot be publicly released due to ethical and privacy restrictions. Additional information regarding data access may be obtained upon reasonable request and with permission from the TSORCC (https://www.tsorcc.org.tw/timecard-formosa-registry).

## Abbreviations

TSORCC: Taiwan society of resuscitation and critical care
TIMECARD: Taiwan management for cardiac arrest multicenter registry
COPD: chronic obstructive pulmonary disease
ESRD: end-stage renal disease
CA: cardiac arrest
IHCA: in-of-hospital cardiac arrest
OHCA: out-of-hospital cardiac arrest
CPR: cardiopulmonary resuscitation
ROSC: return of spontaneous circulation
TTM: targeted temperature management
SBP: systolic blood pressure
DBP: diastolic blood pressure
WBC: white blood cell
INR: international normalized ratio
GCS: Glasgow coma scale scores
cHR: crude hazard ratio
aHR: adjusted hazard ratio
OR: odds ratio
95% CI: 95% confidence interval
RCS: restricted cubic spline
