# Supplementary material for: Effects of Initial Lactate Levels and 24-h Lactate Clearance on Mortality in Post-Cardiac Arrest Patients: Insights from the Multicenter TIMECARD Registry
Source: Int J Med Sci. 2026 Apr 8;23(5):1884–95. doi: 10.7150/ijms.129084 (PMC13133890; doi:10.7150/ijms.129084)

Supplementary material

Figure S1: Restricted cubic spline identifying cutoff values associated with achievement of target lactate clearance rates

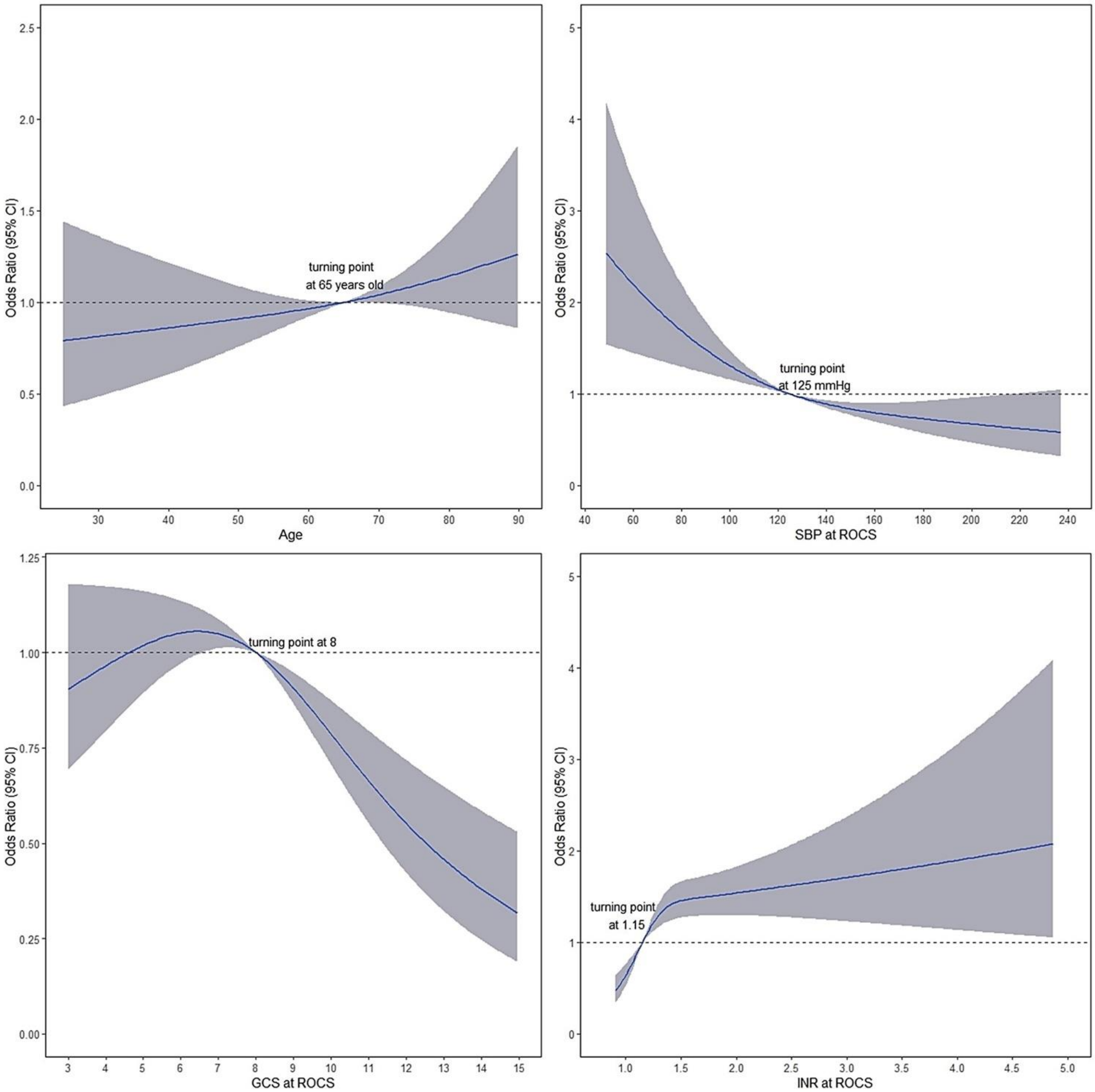

Supplement: Supplementary file 1 — Supplementary figure. [file ijmsv23p1884s1.pdf]
